# Mutual Information-Based Non-Local Total Variation Denoiser for Low-Dose Cone-Beam Computed Tomography

**DOI:** 10.3389/fonc.2021.751057

**Published:** 2021-10-21

**Authors:** Ho Lee, Jiwon Sung, Yeonho Choi, Jun Won Kim, Ik Jae Lee

**Affiliations:** Department of Radiation Oncology, Gangnam Severance Hospital, Yonsei University College of Medicine, Seoul, South Korea

**Keywords:** low-dose cone-beam computed tomography, mutual information, non-local total variation, ray-driven backprojector, low mAs, image-guided radiation therapy

## Abstract

Conventional non-local total variation (NLTV) approaches use the weight of a non-local means (NLM) filter, which degrades performance in low-dose cone-beam computed tomography (CBCT) images generated with a low milliampere-seconds (mAs) parameter value because a local patch used to determine the pixel weights comprises noisy-damaged pixels that reduce the similarity between corresponding patches. In this paper, we propose a novel type of NLTV based on a combination of mutual information (MI): MI-NLTV. It is based on a statistical measure for a similarity calculation between the corresponding bins of non-local patches vs. a reference patch. The weight is determined in terms of a statistical measure comprising the MI value between corresponding non-local patches and the reference-patch entropy. The MI-NLTV denoising process is applied to CBCT images generated by the analytical reconstruction algorithm using a ray-driven backprojector (RDB). The MI-NLTV objective function is minimized based on the steepest gradient descent optimization to augment the difference between a real structure and noise, cleaning noisy pixels without significant loss of the fine structure and details that remain in the reconstructed images. The proposed method was evaluated using patient data and actual phantom measurement data acquired with lower mAs. The results show that integrating the RDB further enhances the MI-NLTV denoising-based analytical reconstruction algorithm to achieve a higher CBCT image quality when compared with those generated by NLTV denoising-based approach, with an average of 15.97% higher contrast-to-noise ratio, 2.67% lower root mean square error, 0.12% lower spatial non-uniformity, 1.14% higher correlation, and an average of 18.11% higher detectability index. These quantitative results indicate that the incorporation of MI makes the NLTV more stable and robust than the conventional NLM filter for low-dose CBCT imaging. In addition, achieving clinically acceptable CBCT image quality despite low-mAs projection acquisition can reduce the burden on common online CBCT imaging, improving patient safety throughout the course of radiotherapy.

## Introduction

Cone-beam computed tomography (CBCT) is used as an image guidance system in many radiotherapy institutions (1). It provides a transformation through registration with a reference image that can be used to adjust the patient’s position to align appropriately with the radiation isocenter, ensuring that the planned dose is accurately delivered to the patient during treatment (2–5). Although there is currently no clinical application of dual-energy CBCT in radiotherapy, with the increase in the use of CBCT imaging devices, the CBCT imaging guidance can potentially be extended to dual-energy capabilities to improve tumor visualization or localization in patients. The only method that is readily adaptable to existing commercial scanners involves two sequential CBCT scans at different tube voltage settings for a high-energy kilovoltage peak and a low-energy kilovoltage peak. Thus, tissues and other materials can be distinguished by exploiting the nature of X-ray attenuation changes in different photon spectra. However, the material decomposition process is highly sensitive to noise fluctuations owing to the overlap of x-ray spectra at low and high energies. Even this approach comes at the expense of doubling radiation dose. Accordingly, the need for patient exposure management is emerging. Particularly, considering the high radiation sensitivity of pediatric cancer patients, early detection of cancer, and increased life expectancy of cancer patients, it is necessary to appropriately manage the imaging dose accompanying image-guided radiation therapy (IGRT), according to the principle of “as low as reasonably achievable”  (6, 7). The report of the American Association of Physicists in Medicine (AAPM) Task Group 75 also emphasizes the need for an imaging dose management in the field of radiation oncology  (8). Of note, the CBCT image dose management has a correlation with the image quality. In general, it is correlated with lower dose values when the milliampere-second (mAs) parameter related to the tube current and exposure time per projection is adjusted toward a lower value (9, 10), resulting in excessive noise in the reconstructed CBCT images. If a clinically acceptable CBCT image quality can be obtained with a lower mAs of the projection data, the risk of radiation exposure to imaging doses is minimized (11). Therefore, it is necessary to develop an algorithm that can improve the CBCT image quality while appropriately managing the indication-oriented patient dose.

CBCT reconstruction generates a volumetric image from the projection data acquired at various angular positions during a single gantry rotation. The reconstruction problem is formulated as a set of mathematical relations considering the scanning geometry between the volume and projections and is solved through various assumptions and strategies. These solutions generally fall into the three broad categories of analytical, iterative, and deep learning approaches. Analytical methods calculate the volume directly from the voxel-driven backward projection transform, describing pixels of projections corresponding to each value of the voxels. Certain types of analytical methods are based on a filtered backprojection (FBP) algorithm involving two main steps, first performing filtering on each set of projection data and then backprojecting the filtered projection data  (12–14). CBCT image quality is biased *via* an advanced denoising technique (10, 15–17) because the performance relies on the filtering operation. The total variation (TV) tended to keep the edge information too smooth by uniformly penalizing the local image gradient (18, 19). Anisotropic TV was introduced to reduce the blurriness at edge regions by deriving an adaptively weighted local image gradient (17). However, these local edge detection operators have limitations in reliably separating structures with low contrast to noise. NLTV was developed to allow for more global searches and non-uniform weights, depending on non-local means (NLM) filter, which accounts for the difference in intensity between pixel pairs (10, 20, 21). This NLM filter can reduce the similarity between corresponding patches when the patches used to determine the pixel weights contain noise-damaged pixels. Conversely, iterative reconstruction methods continually refine the volume to find an optimal solution by repetitively performing forward and backward projection operations. Accurate reconstructions have been realized by integrating physical, statistical, and/or heuristic modeling of a CT system  (22–26). Its efficacy has been improved by incorporating a compressed sensing theory (19, 20, 27). A disadvantage of iterative techniques is that the noise properties are non-linear and unpredictable across the field of view (FOV) as compared to linear noise in analytical methods. Several deep learning-based reconstructions have recently been introduced as supervised training using paired images and unsupervised training using unpaired images  (28–34). Achieving satisfactory results requires sufficient training data. The lack of stability and injectivity of deep learning approaches to image reconstruction poses potential problems that require further investigation (35).

In a previous study, we implemented a conventional non-local total variation (NLTV) based on NLM filter that focused mainly on optimizing the performance of the denoiser on the projection domain (10). However, in general, because the number and resolution of the projections are larger than those of the reconstructed images, the calculation time is longer than when the denoiser is applied in the image domain. Therefore, in this study, we propose a mutual information-based NLTV (MI-NLTV), which is a new type of NLTV applied to the image domain. To our knowledge, this study, integrating MI into NLTV, is the first to augment the image quality of low-dose CBCT generated with a low mAs parameter value. In particular, this approach is effective when used in conjunction with an analytical reconstruction algorithm using a ray-driven backprojector (RDB) (14). The RDB tends to be susceptible to aliasing artifacts but is potentially more accurate because it maintains the exact geometric path of the rays forming the projection data. The algorithm consists of two main steps. The FBP reconstruction algorithm based on RDB is first performed by calculating the length of the intersection between the ray paths and each voxel to be reconstructed. A post-refinement process is subsequently performed using the MI-NLTV denoiser by minimizing the weighted total variation objective function based on the steepest gradient descent optimization with an adaptive step size. This minimization process removes noisy pixels remaining in the reconstructed images and cleans the edges without losing a large amount of fine structure and details. The effectiveness of the proposed method is demonstrated using patient data and actual measurement data based on the Catphan^®^503 and anthropomorphic head-and-neck phantoms.

## Methods

### Ray-Driven Backprojector Based on X-Ray Transmission Length Calculation

A circular pre-weighting factor on each projection was applied to circumvent the intensity drop caused by the cone angle effect  (17). A one-dimensional (1D) ramp filter using the product of the Shepp–Logan filter and raised cosine window function was thereafter applied to repress the high frequency in the frequency domain (10). The ramp filter was multiplied by the Fourier-transformed values from each line parallel to the horizontal direction of the pre-weighted projection *via* 1D Fourier transformation. The filtered projection was obtained by applying an inverse 1D Fourier transformation.

In the proposed approach, the backprojection step is performed after this filtering step to achieve a volumetric image from the filtered projection data. In contrast to the use of a pixel-driven backprojector (PDB) that determines the corresponding location on the detector from the voxels to be reconstructed, in this work, we developed an RDB based on ray tracing (36). This RDB is potentially more accurate than a PDB because it retains the exact geometry of the rays that form the projection data (14). It computes the length of the intersection between the ray paths and each voxel and is expressed as follows:


(1) $$\mu_{j}=\frac{\Sigma_{k}l^{jk}P_{k}}{\Sigma_{k}l^{jk}},$$

where *μ _j_* is the attenuation coefficient to be reconstructed on the *j* th indexed voxel (*V_j_*) in the volume, *P_k_* is the value of the *k-th* indexed pixel in the filtered projection data, and *l^jk^* is a voxel-dependent weighting factor that is the length of the intersection between *V_j_* and the ray originating from *P_k_* (*l^jk^* = 0, if there is no intersection). This ray-tracing process is applied to all the pixels in the projection data. First, all the *μ_j_* values are set to zero. To determine the voxels that affect *P_k_*, a backprojection ray (*α^k^*) that originates from the center position of *P_k_* and travels back to the focal point is considered. The intersections between *α^k^* and the voxels in the volume are subsequently calculated using the Siddon method. At each voxel *V_j_* where *α^k^* passes, the current value of *μ_j_* is increased by l^jk^P_k_. The volume is finally normalized by dividing each *μ_j_* by $\Sigma_{k}l^{jk}.$

### Mutual Information-Based Non-Local Total Variation Denoiser

A weighted total variation denoising process is applied on the reconstructed images to enhance the intensity difference between the striking features and unsolicited noise by determining the similarities between non-local patches (10, 20, 37, 38). When ∅*_i,j_* is the area affected by the center point of each patch, the state of the stationary patch is determined by calculating the statistical measurements with the moving patches included in the search set. The MI (39, 40) is used as a metric of image matching that does not require a linear relationship between the corresponding intensities. If the stationary patch and the moving patch do not match, then the MI has a low value. A stationary patch with a pattern comprising noise-corrupted pixels without noticeable features is considered a high-entropy region. Therefore, it is likely to be in the smoothing state. Conversely, the area to be placed in the preserving state may be a patch with low entropy and a high MI value. Moreover, the MI value is less than or equal to the amount of marginal entropy for each patch. Considering these characteristics, a statistical measure *M*(∅*_i,j_*) is defined as follows:


(2) $$M(\emptyset_{i,j})=\frac{MI(I_{A},I_{B}^{\Omega })}{H(I_{A})}$$

Here, *I_A_* indicates a region in the stationary patch and the search set for the non-local patch, *I_B_* is defined as Ω. $I_{B}^{\Omega }$ implies all the non-local patches included in the search set corresponding to the stationary patch.


(3) $$MI(I_{A},I_{B})=H(I_{A})+H(I_{B}^{\Omega })-H(I_{A},I_{B}^{\Omega }),$$

where H(I_A_)and $H(I_{B}^{\Omega })$ represent the marginal entropy of the stationary patch and moving patch, respectively. These entropies are defined as follows:


(4) $$H(I_{A})=-\Sigma_{i\in I_{A}}p_{i}\log_{2}p_{i},$$

(5) $$H(I_{B}^{\Omega })=-\Sigma_{i\in I_{B}^{\Omega }}p_{i}\log_{2}p_{i},$$

(6) $$H(I_{A},I_{B}^{\Omega })=-\Sigma_{i\in I_{A}}\Sigma_{j\in I_{B}^{\Omega }}p_{i,j}\log_{2}p_{i,j}.$$

Here, *Pi* and *P_i,j_* are the marginal probability distribution and the joint probability distribution of I_A_ and $I_{B}^{\Omega }$ , respectively. For the probability distributions required to calculate these entropies, a joint histogram is used to consider the relationship between the intensities of the corresponding pixels in two stationary and moving patches. The joint histogram is developed by scaling the intensity value of each patch according to the binning size that is adjusted such that the maximum value of each axis of the joint histogram equals the specified value.

**Figure 1:**
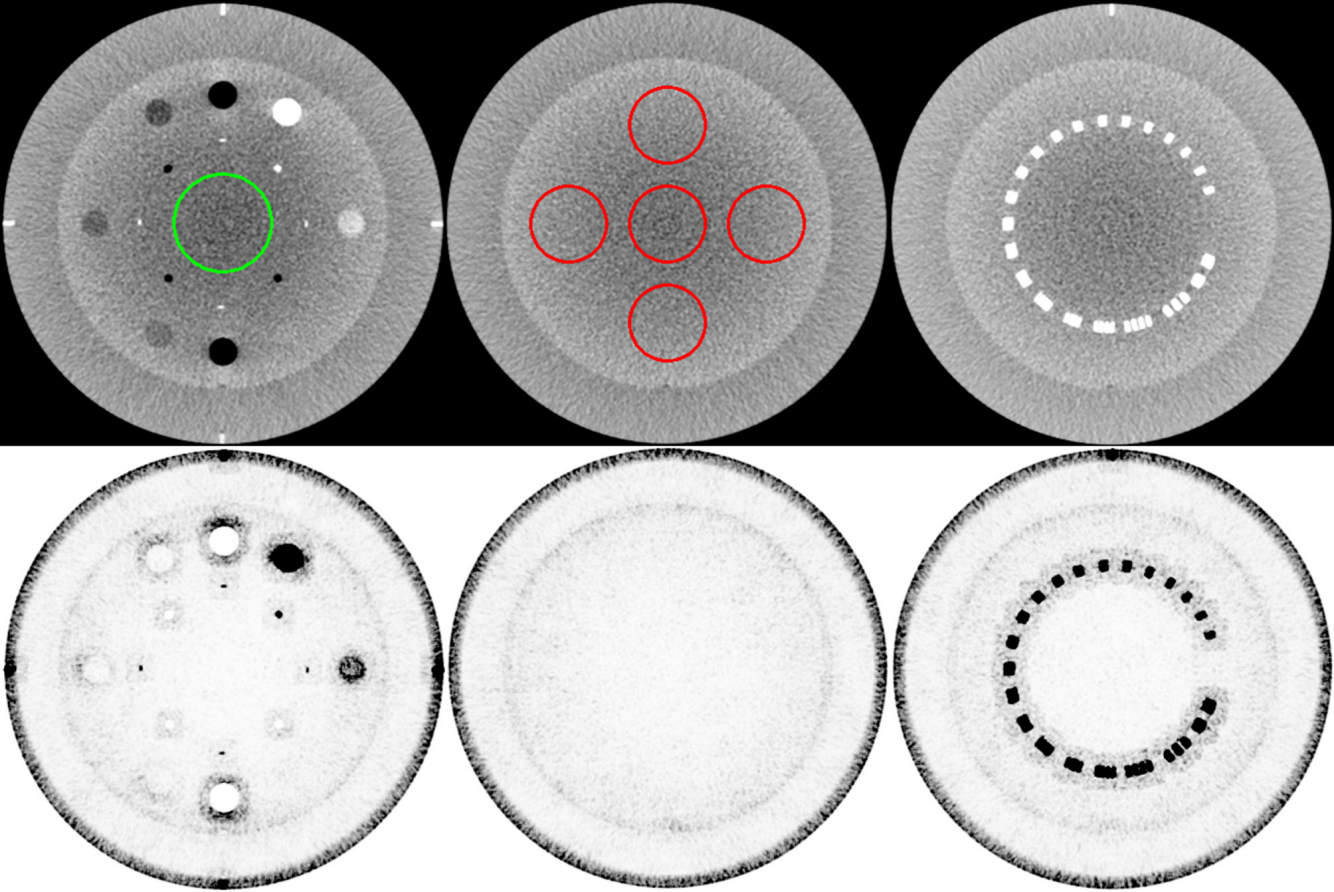
Weight map of the MI-NLTV denoiser that composes the MI-based statistical measure and the spatially encoded factor. The first row shows the original image generated by applying the analytical reconstruction algorithm based on RDB. The ROI in the left image identifies the location of the background for CNR calculation, and the ROI in the middle is for SNU calculation. The second row shows the corresponding weight map. These weight maps are displayed at the window (width and level) settings of (0.2, 0.9).

The penalty with different weights based on *M*(∅*_i,j_*) is expressed as follows:


(7) $$w_{j}=\exp(-{\left(V_{j}/\tau \right)}^{\rho }M(\emptyset_{i,j})),$$

where *j* identifies the index of the voxel element in the image, and *V_J_* is the *j*th voxel element. *w_j_* denotes the weights between the voxels in Ω and the current voxel *j*. A patch size is defined to be (2*a* + 1) × (2*a* + 1) with unit variance and *a* was set to 2, such that the patch size is 5 × 5 in this study. The non-local search extent is 21 × 21 with unit variance. (*V_j_*/τ)^ρ^ is the spatially encoded factor that reduces the weighted averaging effect at high-intensity localities to maintain the contrast (37). The normalization factor τ ensures that the *V_j_*/τ ratio exceeds 1. It was set to 90% of the cumulative distribution function histogram that accumulates the intensity at each voxel of the reconstructed image. The scaling factor γ ensures smaller weights for higher intensities (in this study, ρ = 10). **Figure 1** depicts the MI-NLTV weighting map calculated using Equation (7) on the CBCT image. The dark regions indicate the preservation voxels, and the light regions represent voxels affected by smoothing.

Minimizing the weighted TV objective function in Equation (8) indicates that the edges with high contrast relative to the non-local patches are preserved and that the noisy voxels with low contrast are smoothed.


(8) $$R(V)=\Sigma_{j}R(V_{j})=\Sigma_{j}w_{j}D(V_{j}),$$

(9) D(Vj)=(V(x,y)−V(x−1,y))2+(V(x,y)−V(x,y−1))2,

where *V_(x,y)_* is the voxel element at the 2D position (x, y). The weighted TV objective function is minimized based on the steepest gradient descent method with an adaptive step size that is expressed as follows:


(10) $$V_{j}^{t+1}=V_{j}^{t}-\lambda \nabla R(V_{j})/\left|\nabla R(V)\right|,$$

(11) $$\lambda =\gamma \sqrt{\Sigma_{j}{\left(V_{j}^{t}\right)}^{2}},$$

where λ is an adaptive parameter that reduces the smoothing degree as the iteration step progresses (10). By using the square root of all voxel elements updated in each step, λ is enforced to gradually smaller values with an increase in the number of iterations. To escape local minimization due to sudden changes, a scaling parameter *γ* is used and set to an initial value of 1.0. If *R(V)* in the current iteration step is larger than that in the preceding step, this value is linearly decreased by multiplying a constant value (*r_red_* = 0.8. ∇R(*Vj*) is the gradient of the objective function *R*(*V*) at the *j*th indexed pixel. The root sum square of the gradient calculated at all the pixels, |∇*R*(*V*)|, is required for the normalized gradient calculation. The expression below is provided for clarity.


(12) $$\nabla R(V_{j})=\frac{\partial R(V)}{\partial V_{j}}=\frac{\partial R(V)}{\partial V_{\left(x,y\right)}}=(\begin{array}{c} w_{\left(x,y\right)}\frac{2V_{\left(x,y\right)}-V_{\left(u-1,v\right)}-V_{\left(u,v-1\right)}}{\sqrt{{\left(V_{\left(x,y\right)}-V_{\left(x-1,y\right)}\right)}^{2}+{\left(V_{\left(x,y\right)}-V_{\left(x,y-1\right)}\right)}^{2}}}\\ +w_{\left(x+1,y\right)}\frac{V_{\left(x,y\right)}-V_{\left(x+1,y\right)}}{\sqrt{{\left(V_{\left(x+1,y\right)}-V_{\left(x,y\right)}\right)}^{2}+{\left(V_{\left(x+1,y\right)}-V_{\left(x+1,y-1\right)}\right)}^{2}}}\\ +w_{\left(x,y+1\right)}\frac{V_{\left(x,y\right)}-V_{\left(x,y+1\right)}}{\sqrt{{\left(V_{\left(x,y+1\right)}-V_{\left(x-1,y+1\right)}\right)}^{2}+{\left(V_{\left(x,y+1\right)}-V_{\left(x,y\right)}\right)}^{2}}} \end{array}),$$

(13) $$\left|\nabla R(V)\right|=\sqrt{\Sigma_{j}{\left(\nabla R(V_{j})\right)}^{2}.}$$

The number of iterations is fine-tuned for the gradient descent optimizer. In this study, it was set to 20, which was the optimal number of iterations. The pseudo-code of the MI-NLTV denoiser considers all the abovementioned components and is presented in **Appendix A**.

### Experimental Studies

The Catphan^®^503 phantom (The Phantom Laboratory Inc., Salem, NY) and anthropomorphic phantom CIRS ATOM 701 (Computerized Imaging Reference Systems Inc., Norfolk, VA, USA) were used for CBCT acquisition. The acquisitions were performed on an Infinity™ LINAC system with XVI R5.0 (Elekta Limited, Stockholm, Sweden) in its service mode. The Catphan^®^503 phantom includes different modules for assessing the quality of 3D images. CBCT projection acquisitions were performed with full gantry rotation in a small FOV protocol using the S20 collimator and F0 filter cassettes into the kV source arm. The F0 filter cassette is a blank filter and has no effect on the X-ray beam. The S20 gives a nominal irradiation field width of 27.6 cm at the isocenter. The total number of scan projections was 670 for the Catphan^®^503 phantom and 655 for the anthropomorphic phantom. The size of the X-ray projection at the detector panel was 409.6 mm^2^ × 409.6 mm^2^, containing 1,024 × 1,024 pixels. The source to detector panel distance and the source to axis distance were 1,536 and 1,000 mm, respectively. The low-dose acquisition protocol settings were 100 kVp and 0.1 mAs during each projection. The reconstructed CBCT images of the phantom were generated with 512 × 512 × 100 voxels, comprising 0.5 × 0.5 × 1.0 mm^3^ per voxel. For quantitative comparison, a high-dose CBCT with increased mAs settings (100 kVp and 1.6 mAs) was scanned as a benchmark image. A transformation to the Hounsfield unit (HU) was performed for all the reconstructed CBCT images. The quantifications of image quality were based on contrast-to-noise ratio (CNR), root mean square error (RMSE), spatial non-uniformity (SNU), and correlation (17, 19). Task-based image quality assessments were also performed using an open-source software (imQuest, Duke University, Durham, NC, USA), which includes a task-based transfer function (TTF) for the spatial resolution and a noise power spectrum (NPS) for the noise texture and magnitude (41, 42). The combined results of NPS and TTF were used to evaluate the detectability index (43). For the detectability index calculation, a non-prewhitening matched filter (NPW) and 2D option were used as an observation model and NPS interpolation method, respectively.

The Catphan^®^503 CTP404 module was used for evaluating the correlation, the RMSE, the CNR, and the TTF. A central region of interest (ROI) shown in the left column of the first row in **Figure 1** identifies the location of the background for CNR calculation. The TTF was calculated using a circular ROI around each density insert to derive an edge spread function followed by Fourier Transform. Twenty-one consecutive axial slices including density inserts were selected for analysis. The Catphan^®^503 uniformity module was used for calculating the SNU and the NPS. The ROIs for SNU calculation are shown in the middle column of the first row of **Figure 1**. The 104 ROIs for NPS calculation were selected on 13 consecutive axial slices (8 ROIs/slice × 13 slices = 104 ROIs). Overall frequencies were compared using 1D profiles. To visually compare spatial resolution differences with bin sizes in the joint histogram, profiles were obtained using a resolution gauge in the Catphan^®^503 phantom.

A patient with pelvic bone metastases was also scanned for clinical data collection. The patient projection data were acquired by a Versa HD™ LINAC system with XVI R5.0 in its clinical mode. The S20 collimator, F0 filter, x-ray tube current, pulse duration per projection, and the resolution and voxel size of the reconstructed images were set to the same values as those of the phantom acquisition. In contrast, the voltage of the x-ray tube was set to 120 kVp, and 185 projections were obtained, lower than the number of projections obtained during the phantom scan by a factor of 0.28, including partial scans within 200° instead of a full rotation. The resolution of each projection was 512 × 512 pixels, which was lower than the phantom setting.

## Results

**Figure 2:**
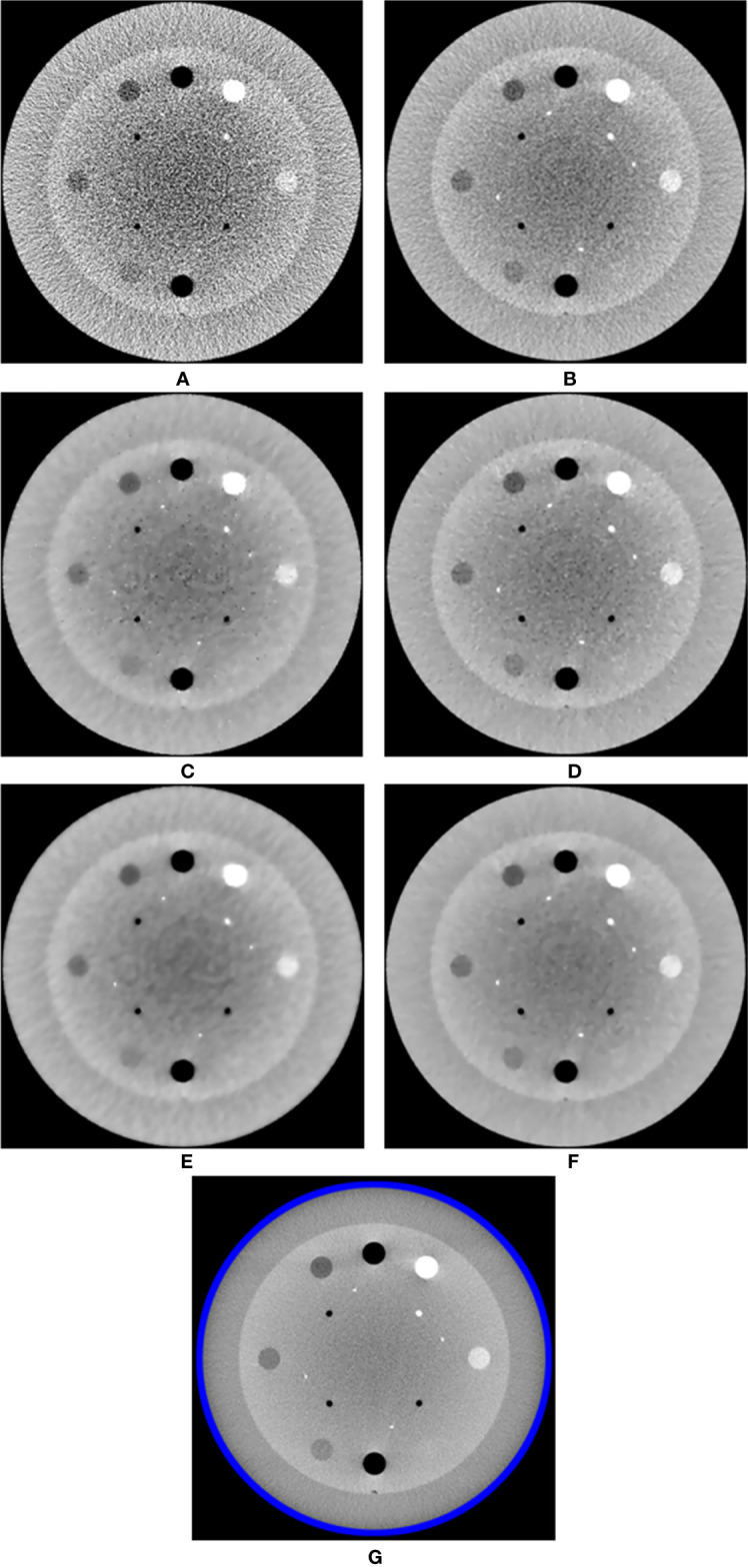
Comparison between the same views of the CBCT images generated by applying the analytical reconstruction algorithm based on **(A)** PDB, **(B)** RDB, **(C)** PDB followed by NLTV, **(D)** RDB followed by NLTV, **(E)** PDB followed by MI-NLTV, and **(F)** RDB followed by MI-NLTV using the Catphan^®^503 phantom. **(G)** Benchmark image. These images are displayed at the window width (975 HU) and the window level (0 HU).

The MI-NLTV denoiser was applied to CBCTs generated by analytical reconstruction algorithms using PDB or RDB based on the Catphan^®^503 phantom. Its performance was compared with that observed in case of the NLTV based on an NLM filter, as well as without a denoiser. **Figure 2** shows a representative slice of the CBCT reconstructed by the analytical reconstruction algorithm based on the PDB without the denoiser and the PDB followed by NLTV and MI-NLTV as well as the RDB without the denoiser and the RDB followed by NLTV and MI-NLTV. The RDB evidently achieved substantial gains when no denoiser was used and when NLTV and MI-NLTV were employed. The artifacts were significantly suppressed in the CBCT images processed by the proposed RDB in conjunction with the MI-NLTV denoiser compared with those processed through other approaches. In particular, the CBCT images followed by the MI-NLTV denoiser were smoother and exhibited fewer artifacts for both PDB and RDB, resulting in slightly lower spatial resolution compared to NLTV.

In addition, we calculated the CNR for each image for all reconstructed CBCTs included in the Catphan^®^503 CTP404 module. The CTP404 module contains seven sensitometry targets made from Delrin™, Teflon, air, polymethylpentene (PMP), low-density polyethylene (LDPE), polystyrene, and air. By selecting a central ROI and seven ROIs within the density target, the mean HU values and standard deviation were recorded to calculate the CNR for each CBCT image. The relative image contrast between the corresponding regions could be compared. **Table 1** provides the average value of the CNRs calculated for each insert in the CBCT images obtained based on the analytical reconstruction algorithms followed by no denoiser, NLTV, and MI-NLTV. **Table 2** provides the statistical significance (p-values) of comparing the CNR values of six combinations to determine whether the CNR values are statistically higher or lower for each type of insert.

**Table 1:** Comparison of contrast-to-noise ratio (CNR) values at seven ROIs in the CBCT image generated by analytical reconstruction algorithms based on Pixel-driven backprojector (PDB) and Ray-driven backprojector (RDB) using low-dose projection data of the Catphan^®^503 phantom.

ROI	Material of insert	PDB	RDB	PDB+non-local total variation (NLTV)	RDB+NLTV	PDB+mutual information (MI)-NLTV	RDB+MI-NLTV
1	Delrin™	2.5	10.5	17.2	21.1	25.3	25.8
2	Teflon	5.6	19.4	28.7	31.2	28.4	29.7
3	Air	5.9	27.1	40.3	51.8	44.1	61.6
4	Polymethylpentene	0.6	2.8	4.3	6.4	6.2	8.2
5	Low-density polyethylene	0.1	0.6	0.8	1.3	1.2	1.7
6	Polystyrene	0.3	1.0	2.4	2.2	3.8	2.9
7	Air	5.9	27.9	43.2	54.4	54.7	65.4

**Table 2:** Statistical analysis comparing the CNR values of RDB+MI-NLTV vs. different combinations and PDB+MI-NLTV vs. PDB+NLTV for each type of insert using the paired t-test.

p-value	Delrin™	Teflon	Air	Polymethylpentene	Low-density polyethylene	Polystyrene	Air
RDB+MI-NLTV vs. PDB	0.000	0.000	0.000	0.000	0.000	0.000	0.000
RDB	0.000	0.000	0.000	0.000	0.000	0.000	0.000
PDB+NLTV	0.000	0.432	0.000	0.000	0.000	0.000	0.000
RDB+NLTV	0.000	0.131	0.000	0.000	0.000	0.000	0.000
PDB+MI-NLTV	0.427	0.249	0.000	0.000	0.005	0.000	0.000
PDB+MI-NLTV vs. PDB+NLTV	0.000	0.646	0.001	0.000	0.000	0.000	0.000

The NLTV and MI-NLTV show improved CNRs in all ROIs when compared with those obtained with PDB or RDB without a denoiser. Compared with PDB+NLTV and RDB+NLTV, PDB+MI-NLTV and RDB+MI-NLTV showed higher CNRs in all ROIs except for those of Teflon. In the case of Teflon, which has the highest HU of the seven inserts, MI-NLTV combinations showed statistically insignificant results because the p-values were greater than 0.05 when compared with the NLTV combinations. The reason is that both NLTV and MI-NLTV use spatially encoded factors to maintain high-intensity contrast in Teflon. Although combining RDB in MI-NLTV yielded slightly higher CNR values than combining PDB in MI-NLTV (except for polystyrene), these values are considered statistically similar.

**Figure 3:**
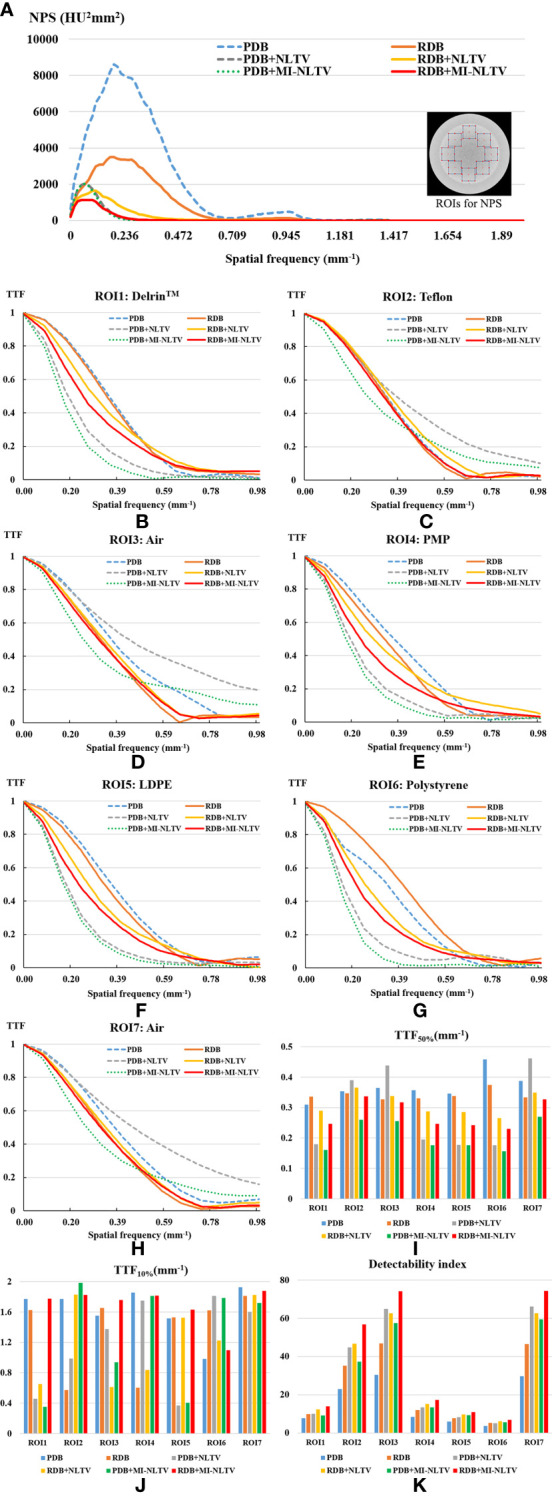
Task-based image quality assessment for PDB, RDB, PDB+NLTV, RDB+NLTV, PDB+MI-NLTV, and RDB+MI-NLTV. **(A)** Noise power spectrum (NPS) for evaluating the noise texture and magnitude. **(B–H)** The task-based transfer function (TTF) curves calculated by selecting a circular ROI around each insert among seven different density targets. **(I)** TTF_50%_ values and **(J)** TTF_10%_ values for assessing the spatial resolution. **(K)** The detectability index based on NPS and TTF for estimating the ability to detect some regions.

**Figure 3** shows the NPS curves and the TTF curves at seven density inserts for all reconstruction types. The eight square ROIs were placed at different positions in the Catphan^®^503 uniformity module, as shown on the right side in **Figure 3A**. The NPS peaks were lower for all RDB types than their corresponding PDB types. The lowest NPS peak value was obtained as 1,139 HU^2^mm^2^ for RDB+MI-NLTV. The NPS average spatial frequency shifted to lower frequencies when combining NLTV or MI-NLTV with PDB or RDB. Numerically, the NPS average spatial frequencies were obtained as 0.29 mm^-1^ for PDB, 0.27 mm^-1^ for RDB, 0.13 mm^-1^ for PDB+NLTV, 0.17 mm^-1^ for RDB+NLTV, 0.11 mm^-1^ for PDB+MI-NLTV, and 0.12 mm^-1^ for RDB+MI-NLTV. In terms of spatial resolution, TTF values tended to decrease as the noise magnitude decreased for all ROIs. In particular, it should be noted that MI-NLTV denoiser did not appear to enhance the whole spatial resolution while reducing noise magnitude. In the comparison of MI-NLTV and NLTV, TTF_50%_ values with MI-NLTV denoiser were lower than with NLTV denoiser for both PDB and RDB. Conversely, TTF_10%_ values showed that MI-NLTV was more dominant than NLTV. As such, similar spatial resolutions were found between MI-NLTV and NLTV when considering TTF_50%_ and TTF_10%_. However, the combined results of the NPS and the TTF showed that the detectability index of the seven density inserts was higher with RDB+MI-NLTV than with other reconstruction types. This means that the CBCT image quality can be improved with RDB+MI-NLTV because the noise reduction effect is much greater than the spatial resolution reduction.

**Table 3** provides three quantitative measures (RMSE, correlation, and SNU) obtained from the CBCT images generated based on six combinations. After calculating the RMSE, correlation, and SNU for each image of all reconstructed slices included in the Catphan^®^503 module section, the average values were recorded. A paired t-test was also used to determine whether the paired measurements of the proposed and alternative combinations were statistically significant. The p-values obtained by the paired t-test are listed in **Table 4**.

**Table 3:** Quantitative comparisons based on three metrics in the CBCT image generated by six analytical reconstruction algorithms using low-dose projection data of the Catphan^®^503 phantom.

	PDB	RDB	PDB+NLTV	RDB+NLTV	PDB+MI-NLTV	RDB+MI-NLTV
Root mean square error (RMSE)	145.3	59.3	54.4	53.6	55.9	52.1
Correlation	0.51	0.84	0.87	0.88	0.89	0.89
Spatial non-uniformity (SNU)	11.45	10.08	10.58	9.97	11.27	9.96

**Table 4:** Statistical analysis of the three metrics of RDB+MI-NLTV and five approaches using a paired t-test.

		RDB+MI-NLTV vs.				
		PDB					RDB	PDB+NLTV	RDB+NLTV	PDB+MI-NLTV
p-value	RMSE	0.000	0.000	0.000	0.000	0.000
	Correlation	0.000	0.000	0.000	0.000	0.770
	SNU	0.000	0.000	0.000	0.006	0.000

The RMSE was calculated to assess the differences in the reconstructed and benchmark images, whereas the correlation evaluates the concordance with the benchmark image. For the RMSE and correlation measurements, a circular measurement region completely inside the phantom was chosen, as shown in **Figure 2G**; the surrounding air region of the phantom was excluded. The SNU was also calculated by selecting five ROIs with uniform intensity distribution on the Catphan^®^503 uniformity module, one being located in the center and four in peripheral positions symmetrically arranged around the center. The RDB could yield a lower RMSE, higher correlation, and lower SNU compared with the PDB. Applying either the NLTV or MI-NLTV denoiser produced better results regardless of employing the PDB or RDB for the Catphan^®^503 phantom for a low-dose scenario. The correlation values varied widely between PDB and RDB but were close after the NLTV or MI-NLTV denoisers were applied. When MI-NLTV was applied to PDB, the recorded correlation was always better and the recorded RMSE and SNU were worse than when NLTV was applied, whereas RDB followed by the MI-NLTV denoiser produced the lowest RMSE, highest correlation, and lowest SNU. Except for the correlation between the RDB followed by NLTV vs. the proposed method, there was statistical significance between the proposed method and five combinations because all p-values were less than 0.05.

**Figure 4:**
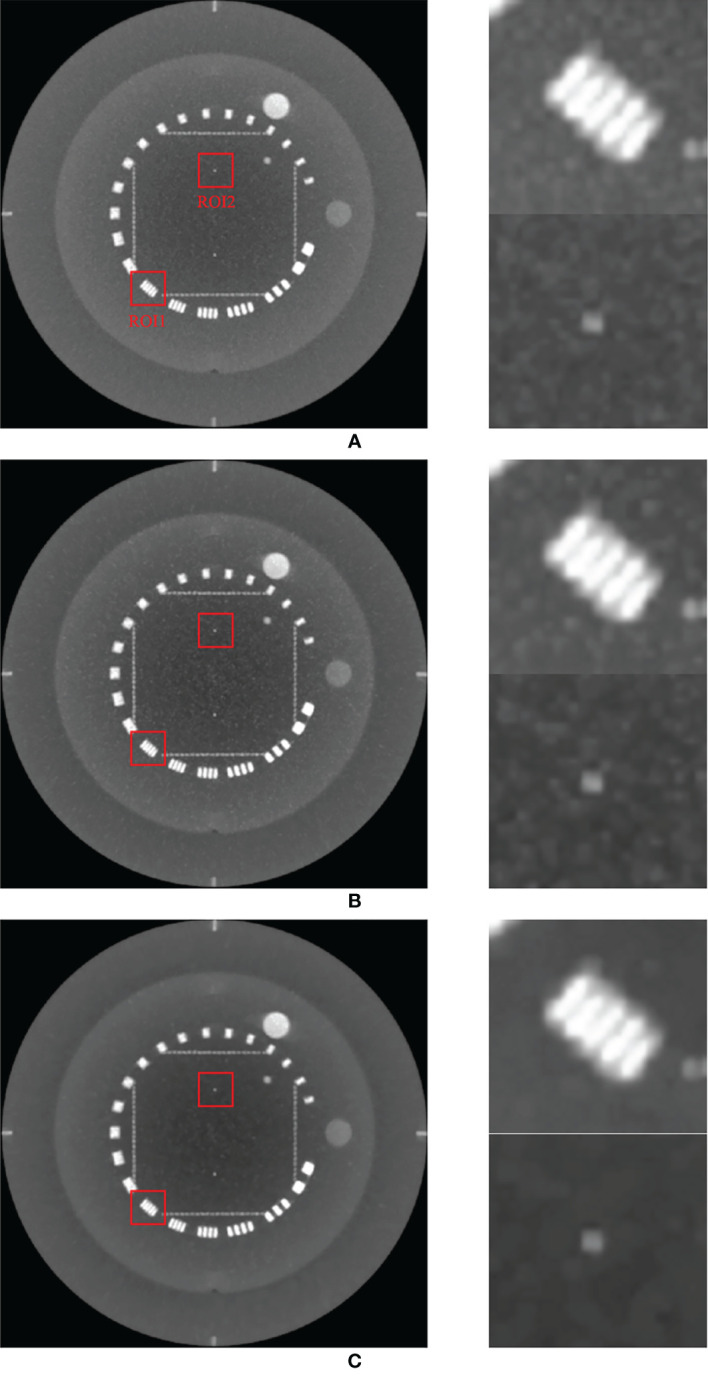
Comparison of maximum intensity projections (MIPs) of the CBCT images generated by applying analytical reconstruction algorithm based on **(A)** RDB, **(B)** RDB followed by NLTV, and **(C)** RDB followed by MI-NLTV using the Catphan^®^503 phantom. These images are displayed at the window (width and level) settings of (1,500, 500) HU.

**Figure 4** compares the maximum intensity projections (MIPs) of the low-dose CBCT images reconstructed by the RDB without denoiser, RDB followed by NLTV, and RDB followed by MI-NLTV. It consists of projecting the voxel with the highest value onto a 2D image, traveling the viewing ray in a longitudinal direction throughout the volume. The MIP image enables it to determine whether some regions with high contrast are preserved while reducing the noise. The proposed MI-NLTV method shows that the regions with remarkable features are well-preserved while reducing noisy pixels compared with the results obtained using only RDB as well as with NLTV incorporated. After incorporating the proposed MI-NLTV denoiser, the homogeneous region was smoother, whereas a point feature and line bars showing a high contrast in the red ROIs were almost preserved.

To support our claim that the MI-NLTV denoiser improves the image quality, we calculated the mean HU for the two red boxes in **Figure 4** to provide a quantitative comparison of the image quality of MIP. For ROI1 and ROI2, the mean HU and standard deviation were calculated as 346 ± 365 HU and 91 ± 38 HU for RDB, 312 ± 379 HU and 46 ± 42 HU for RDB+NLTV, and 291 ± 383 HU and 12 ± 30 HU for RDB+MI-NLTV, respectively. The mean HU decreased as expected because the noisy pixels were reduced, whereas the high-contrast pixels were preserved.

**Figure 5:**
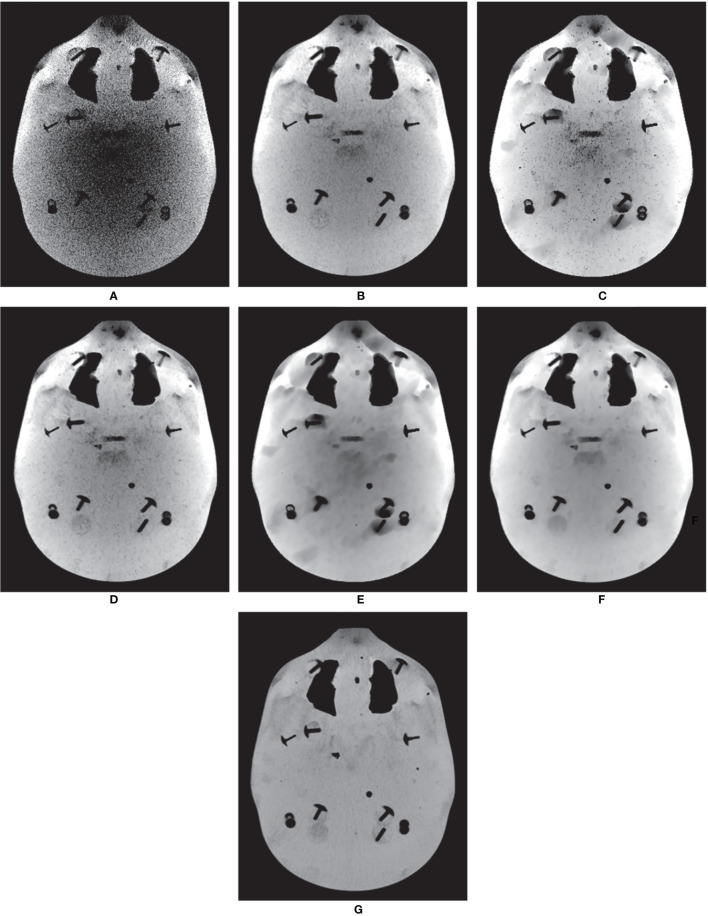
Comparison of minimum intensity projections (MinIPs) of CBCT images generated by applying the analytical reconstruction algorithm based on **(A)** PDB, **(B)** RDB, **(C)** PDB followed by NLTV, **(D)** RDB followed by NLTV, **(E)** PDB followed by MI-NLTV, and **(F)** RDB followed by MI-NLTV using the anthropomorphic head-and-neck phantom. **(G)** Benchmark image. These images are displayed at the window settings of (width = 750, level = 0) HU.

**Figure 6:**
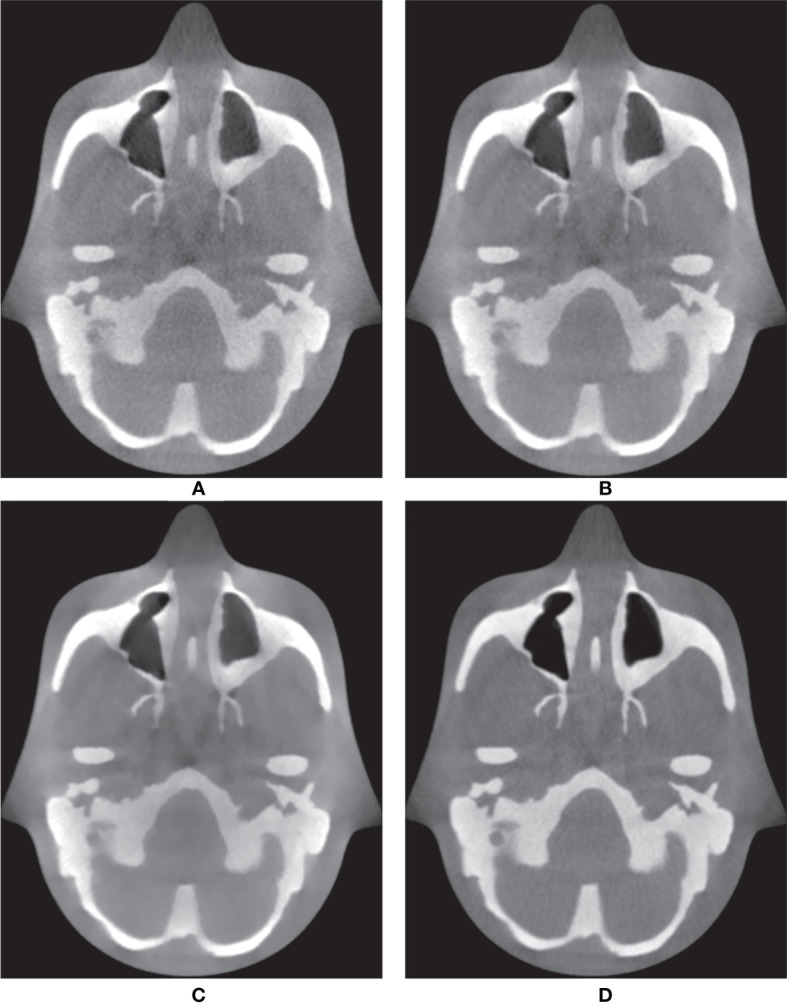
Comparison between the same views of CBCT images generated by applying the analytical reconstruction algorithm based on **(A)** RDB, **(B)** RDB followed by NLTV, and **(C)** RDB followed by MI-NLTV using the anthropomorphic head-and-neck phantom. **(D)** Benchmark image. These CBCT images are displayed at the window settings of (width = 1,400, level = 200) HU.

In addition to the Catphan^®^503 phantom, the anthropomorphic head-and-neck phantom was reconstructed based on six combinations. Because the anthropomorphic phantom is surrounded by the skull, MIP mainly displays voxels contained within the skull, which are composed of high intensities. Therefore, instead of the MIP, **Figure 5** shows the minimum intensity projections (MinIPs) of the reconstruction images along the longitudinal direction. MinIP is the opposite of MIP and returns the lowest value among the voxels that it encounters along the ray. MinIP indicates whether the noisy pixels generated in areas other than the skull corrupt the striking structures. Adding the proposed MI-NLTV denoiser produced improved preservation of the details and reduced the noise markedly, whereas the FBP with PDB was almost completely obscured by noisy pixels. Although employing MI-NLTV remarkably reduced the noise, it was not able to completely restore the detail level of the structures compared with the RDB. **Figure 6** compares the corresponding slices of the low-dose CBCT images reconstructed by the RDB without denoiser and the RDB followed by NLTV and MI-NLTV. MI-NLTV denoiser further reveals improved detail-preserving and noise-reduction effects compared with the NLTV denoiser.

For the quantitative comparison, the CNR was calculated for each image of all the reconstructed CBCTs included in the skull. By selecting an ROI within the soft tissue and an ROI within the skull, the mean HU value and standard deviation were recorded for the CNR calculation. **Table 5** provides the average values of the CNRs calculated for each image in the CBCTs obtained based on RDB followed by no denoiser, NLTV, and MI-NLTV. Statistical testing was also performed to compare the outcomes of the different approaches. The RDB+MI-NLTV combination showed a higher CNR and statistically significant result because the p-value was less than 0.05 when compared with RDB as well as RDB+NLTV.

**Table 5:** CNR comparisons in the CBCT image generated by three RDB-based reconstruction algorithms using low-dose projection data of the anthropomorphic phantom.

		RDB	RDB+NLTV	RDB+MI-NLTV
CNR (mean ± standard deviation)		14.7 ± 3.9	16.7 ± 4.3	19.1 ± 5.5
p-value	RDB+MI-NLTV vs.	0.000	0.000	–

**Figure 7:**
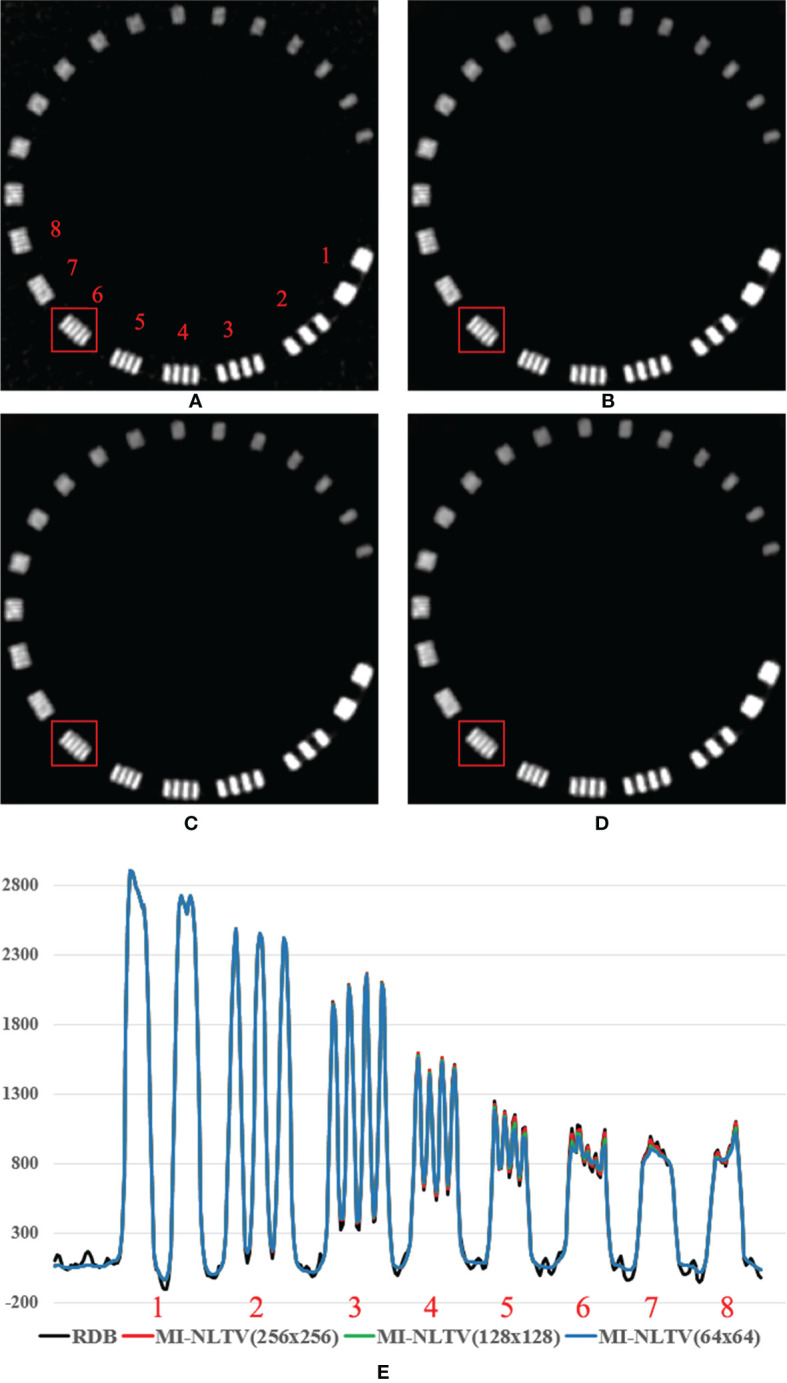
Comparison between the same views of the reconstruction image generated by **(A)** RDB only and RDB followed by MI-NLTV applying **(B)** joint histogram bin size of 256 × 256, **(C)** 128 × 128, **(D)** 64 × 64, and **(E)** line profile at the red square using the Catphan^®^503 phantom. These images are displayed at the window settings of (width = 1,500, level = 876) HU.

To further illustrate the edge information, the spatial resolution was measured using a resolution gauge in the Catphan^®^503 phantom. **Figure 7** shows the 1D HU profile along the orthogonal direction of line bars from 1 through 8 line pairs per cm on the reconstructed images generated by only RDB and RDB followed by MI-NLTVs, with different bin sizes (256 × 256, 128 × 128, and 64 × 64). In the cases of 128 × 128 and 256 × 256 bin sizes, it was possible to identify up to six line pairs/cm due to preservation of major features such as for RDB. Moreover, when using the 64 × 64 bin size, there was a slight difference from six line pairs/cm onward compared with the other bin sizes. To balance the reconstructed image quality with the computational demands, the bin size of the joint histogram was set as 128 × 128 pixels to afford more interesting results.

**Table 6** compares the computation times when generating low-dose CBCT images of the Catphan^®^503 and anthropomorphic phantoms based on six combinations. Each algorithm was implemented by utilizing OpenMP for parallelization on an Intel Xeon CPU system with 48 logical processors on 24 physical cores. Compared with PDB, the calculation time using RDB was approximately 3.7 times longer for the low-dose CBCT generation. Approximately 100 s were required for NLTV and 240 s for MI-NLTV. Because MI generates a joint histogram as a similarity measure between non-local patches, the calculation time was 2.4 times higher than that of the case of a conventional NLTV using NLM filter. Therefore, the computation time using the proposed RDB as well as MI-NLTV was approximately 6.1 times and 1.7 times longer compared with PDB and RDB. **Table 7** delineates the computation time of the proposed MI-NLTV in terms of the joint histogram with different bin sizes. The calculations of the marginal entropy and joint entropy required for MI computation are dependent on the bin size of the joint histogram. The computation time for the MI-NLTV with 128 × 128 bin sizes was half that of 256 × 256 bin sizes.

**Table 6:** Computation time (s) when generating CBCTs of the Catphan^®^503 and anthropomorphic phantoms based on six combinations.

	PDB	RDB	PDB+NLTV	RDB+NLTV	PDB+MI-NLTV	RDB+MI-NLTV
Catphan^®^503	96.5	354.1	196.8	452.8	335.4	586.4
Anthropomorphic	98.7	348.0	196.1	448.3	332.8	589.5

**Table 7:** Computation time (s) when applying MI-NLTVs with different bin sizes on low-dose CBCTs of the Catphan^®^503 and anthropomorphic phantoms.

	MI-NLTV (BIN64×64)	MI-NLTV (BIN128×128)	MI-NLTV (BIN256×256)
Catphan^®^503	171.1	232.4	584.2
Anthropomorphic	173.0	241.5	585.8

**Figure 8:**
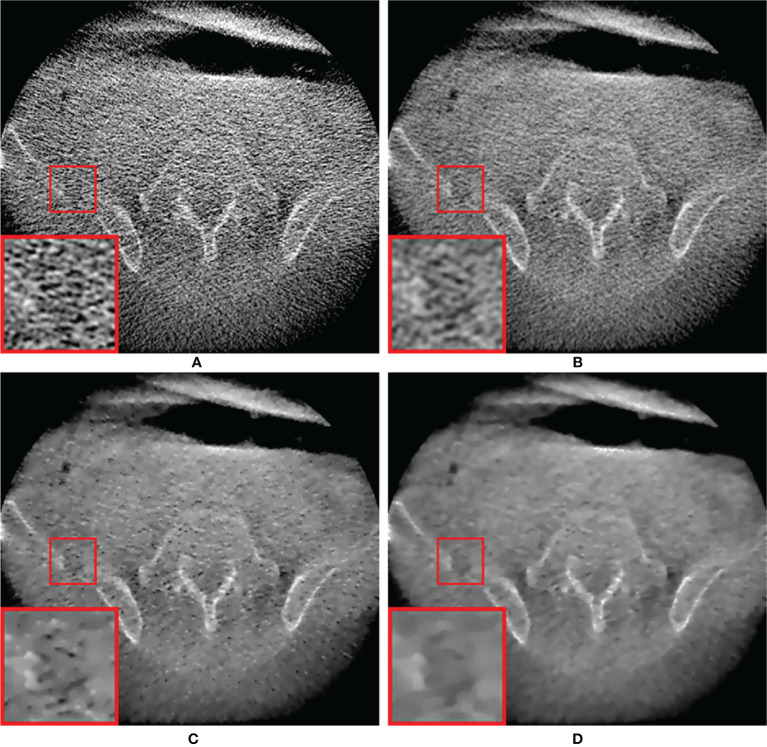
Comparison between the same views of CBCT images generated by applying the analytical reconstruction algorithm based on **(A)** PDB, **(B)** RDB, **(C)** RDB followed by NLTV, and **(D)** RDB followed by MI-NLTV using a patient data with the pelvic bone metastatic lesion. These CBCT images are displayed at the window settings of (width = 2,400, level = 400) HU.

**Figure 8** shows low-dose CBCT images generated from real patient data with pelvic bone metastases. Because the patient projections were acquired under a short scan mode, a short scan weighting in all projections was added to avoid discontinuous artifacts due to redundant scans at certain angles by modifying the Parker’s weighting used in fan-beam CT reconstructions  (44). Compared to the phantom study, the low signal-to-noise level resulted in an overall increase in noise in the reconstructed image and inferior feature details due to the use of a small number of projection data and lower resolution projection data. However, the relative superiority of the proposed MI-NLTV denoiser was also the same in clinical data. PDB was poor at delineating the pelvic bone metastatic lesion due to increased noise, as shown in the red ROI. This lesion appeared as a noisy but faint transparency with the RDB. It was more visible on NLTV as poorly defined, having low intensity with some noise. Meanwhile, the proposed MI-NLTV denoiser could better recognize metastatic bone lesions with fewer noisy pixels than NLTV. It was confirmed that the proposed method can significantly reduce noise pixels and preserve the detailed structure well in patient data. As such, image processing approaches for noise removal and contrast enhancement are of great interest. Noise and low contrast have a direct impact on the delineation of various tumor regions, including enhancing and non-enhancing tumors, necrosis, and edema. They not only affect the ROI extraction, but also interfere with operation in various post-processing tasks such as registration, segmentation, and classification. Therefore, if the quality of low-dose CBCT images is improved through the proposed algorithm, more precise monitoring of the tumor target and movement of the organs at risk (OARs) is possible during IGRT for cancer patients, thereby improving the target accuracy and reducing the dose to the OARs adjacent to the tumor. Furthermore, recalculating the dose distribution for the tumor target and adjacent OARs using the obtained low-dose CBCT may establish a new clinical guideline. This is expected to result in a lower complication rate and improved overall survival rates for radiotherapy outcomes.

## Discussion

This study has aimed to address the feasibility of obtaining low-dose CBCT images using a combination of RDB and MI-NLTV denoiser. The results of this study demonstrate the enhanced performance of the proposed approach compared with the results of the five combinations (PDB, RDB, PDB+NLTV, RDB+NLTV, and PDB+MI-NLTV) for low-dose CBCT. The evaluation utilized is based on a comparison through the image quality phantom analysis. It is demonstrated that in the actual measurement data with the Catphan^®^503 and anthropomorphic head-and-neck phantoms, combining the RDB enables the MI-NLTV denoising-based analytical reconstruction algorithm to be further enhanced. Thereby, a higher CBCT image quality with a lower mAs acquisition protocol is obtained in terms of visual inspection, CNR, RMSE, correlation, SNU, and detectability index. In addition, when using the MI-NLTV denoiser, the spatial resolution was slightly further reduced, but over-smoothing and loss of important features did not occur. Conversely, some noisy pixels remained when the conventional NLTV was employed. In particular, reducing the number of projections by increasing the gantry rotation speed to reduce the patient radiation dose is challenging to implement in the currently available commercial CBCT scanners, whereas the other method proposed in our work, involving lowering the mAs level, can be easily applied in existing commercial scanners.

The proposed MI-NLTV denoiser in the reconstruction process involves a distinct contrast compared with the conventional NLTV approaches using the weight of an NLM filter to account for the difference in intensity between the pixel pairs. The NLM filter degrades the performance in low-dose CBCT images generated with low mAs because the local patch used to determine the pixel weights contains noisy-damaged pixels that reduce the similarity between the corresponding patches. On the contrary, the MI can facilitate overcoming this shortcoming by utilizing a statistical measure for the robust similarity calculation between the corresponding non-local patches and the reference patch. It is almost invariant to the change in noisy pixels, making the NLTV more stable and robust than the NLM filter.

Because the acquisition of CBCT scans utilizes a large-area detector, deterioration in CBCT image quality due to beam scattering is inevitable. However, although this study included improvement in low-dose CBCT image quality *via* the proposed method, measurement-based scatter correction such as an anti-scatter grid (45) or beam blocker (19, 46) was not applied to the acquired CBCT projection data. Considering that scatter correction methods can be used to acquire CBCT projection data, further reductions in the SNU of low-dose CBCT image quality can be expected.

There is no consensus on the guidelines for noise reduction in CBCT images. However, in radiotherapy, CBCT, taken before or during patient treatment, is used to monitor interfraction or intrafraction differences in positional settings and anatomical changes (3). Based on the bone anatomy or soft tissue at the treatment site, rigid body image registration of CBCT and Plan-CT is performed to obtain an appropriate transformation vector to correct motion variations. Recently, it has also been used for deformable image registration between CBCT and Plan-CT for adaptive radiotherapy. The accuracy of this image registration is affected by the difference in image quality of the CBCT used. A prior study reported that higher image quality reduced the uncertainty of soft tissue image registration during IGRT (47). As such, high-quality CBCT images can improve the accuracy of image registration, thereby reducing the target margin during treatment planning. Therefore, reducing the noise level in the low-dose CBCT images provides the best visualization of bone and soft tissue structures, which reduces the uncertainty of image registration during IGRT. In clinical practice, an image processing technique with improved performance such as an MI-NLTV denoising algorithm to remove noise while maintaining the edge structure is needed.

The proposed MI-NLTV denoiser showed better quantitative and qualitative tendencies when combined with RDB instead of PDB. Because this method was applied in the image domain, applying a backprojector that can reduce the noticeable noise level in CBCT image generation appears to produce more stable weight values in MI-based statistical calculations. Instead of RDB, the proposed MI-NLTV can also be combined with other more sophisticated backprojector methods such as distance-driven methods (48) or separable footprints (49). When the MI-NLTV denoiser was applied to CBCT images generated from PDB, obtaining a robust weight value was difficult in the MI-based statistical calculation because the noise level of PDB was relatively high. If projection images could be acquired using slightly higher mAs to bring the NPS peak down to the RDB level, the MI-NLTV denoiser is expected to work well with PDB. Although the proposed MI-NLTV denoiser method was applied to a 2D inter-patched image, the method can be technically extended to 3D blocks (50). This would increase the computational cost because of the larger search area in the weight computations.

This study on low-dose CBCT reconstruction involves a few practical considerations that are discussed here. First, considering the time consumed during the proposed reconstruction approach, the time consumed by the RDB and MI-NLTV denoiser in the experiments is measured. The computation time of the PDB is affected by the reconstructed volume size because it estimates the projection pixel for each voxel in the reconstructed volume. Moreover, the RDB is affected by the number of projections and the size as well as the reconstruction volume size because it uses each pixel of the projection as the starting point of the ray and calculates the intersection length between the ray path and each voxel in the reconstructed volume. The MI-NLTV denoiser increases the computational cost owing to the generation of a joint histogram as a similarity measure between non-local patches when computing the weight function compared with the NLTV denoiser. These algorithms were implemented using OpenMP-based parallelization. The RDB process was parallelized in terms of multiple rays, and the MI-NLTV was mainly parallelized in multiple voxels. It should be noted that there is still a considerable potential for further accelerating the algorithm. A scalable approach would be to improve the computational ability using a GPU (14) or FPGA (51). Second, the parameter values pertaining to the number of iterations, search area, patch size, and spatially encoded factor were set to be equal for ensuring a fair comparison between MI-NLTV and NLTV. These parameters were empirically determined to balance the image quality with the computational load; however, the best performance of either algorithm is not ensured. Nonetheless, we found that the results are not highly sensitive to these parameters. The findings here are not far from optimal. Third, in the deep learning approaches, there are many data augmentation strategies to add more training data based on image processing techniques. The proposed MI-NLTV denoiser can be considered as an advanced augmentation technique for building better statistical models. Fourth, MI-NLTV-based analytical reconstruction algorithm can be used to generate an initial guess image or an image fidelity term for iterative reconstruction. In general, when using an iterative reconstruction algorithm, it has been observed that the better the initial guess image, the faster the convergence and the higher the contrast of the CBCT images produced.

## Conclusion

The incorporation of MI has proven to be almost invariant to the change in the noisy pixels while maintaining the original advantages with similar properties of the conventional NLTV denoiser, making the NLTV more stable and robust than the conventional NLM filter. These differences indicate a preference for the MI in NLTV for low-dose CBCT imaging. Moreover, achieving clinically acceptable CBCT image quality despite low-mAs projection acquisition can reduce the burden on common online CBCT imaging, such as correcting setup errors and monitoring patient movements, thus making the use of IGRT widely available. The proposed approach can improve patient safety throughout the course of radiotherapy.

## Data Availability Statement

The original contributions presented in the study are included in the article/supplementary material. Further inquiries can be directed to the corresponding author.

## Ethics Statement

Ethical approval for this study was obtained from the institutional review board of Yonsei University Health System, Gangnam Severance Hospital (Approval No.: 3-2020-0464). Written informed consent for participation was not required for this study in accordance with the national legislation and the institutional requirements.

## Author Contributions

Conceptualization, HL and IL. Methodology, HL. Software, HL. Validation, IL and HL. Formal analysis, HL. Investigation, HL. Data acquisition, JS and YC. Data curation, JS and YC. Patient enrollment, JK. Writing—original draft preparation, HL. Writing—review and editing, IL. Supervision, IL. All authors contributed to the article and approved the submitted version.

## Funding

This research was supported by the Basic Science Research Program through the National Research Foundation of Korea funded by the Ministry of Education (grant number 2019R1I1A1A01062157) and a grant of the Korea Health Technology R&D Project through the Korea Health Industry Development Institute (KHIDI) funded by the Ministry of Health & Welfare, Republic of Korea (grant number HI19C1330).

## Conflict of Interest

The authors declare that the research was conducted in the absence of any commercial or financial relationships that could be construed as a potential conflict of interest.

## Publisher’s Note

All claims expressed in this article are solely those of the authors and do not necessarily represent those of their affiliated organizations, or those of the publisher, the editors and the reviewers. Any product that may be evaluated in this article, or claim that may be made by its manufacturer, is not guaranteed or endorsed by the publisher.

## Appendix A

*For n*  1 *to all images do*

 *r*  1, *r_red_*  0.8, ε  3, *a*  2, Ω  10

 *For j* 1 *to all voxels do*

  *Calculate D*(*V_j_*) *using Eq.* (9)

  *Create intensity CDF histogram using V_j_*

 *End For*

 τ  *Intensity at* 90% *of intensity CDF*

 *R*(*V*) 0

 *For j*  1 *to all voxels do*

  *w_j_*  0

  *For k*  *a to a do*

   *Find the largest number in V_j_*_+_ *_k_ and call it* ′*A_max_* ′

  *End For*

  *For i*  *j* – Ω *to j* + Ω *do*

   *S*  0

   *For k*  *a to a do*

    *Find the largest number in V_i_*_+_ *_k_ and call it* ′*B_max_* ′

   *End For*

   *For k*  *a to a do*

    *A*  (*V_j+k_*/*A_max_*) × *B in Size*

    *B*  (*V_i+k_*/*B_max_*) × *B in Size*

    *Voting at Bin of (A,B) of the joint histogram*

   *End For*

  *End For*

  *Calculate w_j_ using Eq. (7)*

  *Calculate D*(*V_j_*) *using Eq. (9)*

  *R*(*V_j_*)  *w_j_D*(*V_j_*)

  *R*(*V*)  *R*(*V*) + *R*(*V_j_*)

 *End For*

 *For t*  *to* 20 *do*

  *For j*  1 *to all voxels do*

   λ  $\sqrt{\sum_{j}V_{j}^{2}}$ λ  λ × *r*

   ∂*V_j_* ← ∇*R*(*V_j_*) *calculated by Eq.* (12)

   
|∇R(V)| ← $\sum \sqrt{{\sum_{j}\left(\partial V_{j}\right)}^{2}\;}as\;in\;Eq.\;(13)$

  *End For*

  *For j*  1 *to all voxels do*

   
$V_{j}^{\prime }$  $\sum V_{j}+\lambda \partial V_{j}/\left|\nabla R(V)\right|$

  *End For*

  *While R* $\left(V_{j}^{\prime }\right)$ *> R*(*V*) *do*

   *r*  *r* × *r_red_*

   λ  λ × *r*

   *For j*  1 *to all voxels do*

    
$V_{j}^{\prime }$  $V_{j}+\frac{\lambda \partial V_{j}}{\left|\nabla R(V)\right|}$

   *End For*

  *End While*

  
$Update\;V_{j}^{\prime }\;to\;V_{j}$

 *End For*

*End For*
